# Advancements in Laser-Processed Functional Surfaces for Medical Devices: A Current Review

**DOI:** 10.3390/nano15130999

**Published:** 2025-06-27

**Authors:** Ziyi Xu, Yanxiao Austin Wang, Vivian Ng, Hongyan Yin, Shuai Xu

**Affiliations:** 1 Department of Biomedical Engineering, College of Design and Engineering, National University of Singapore, Singapore 117583, Singapore; ziyixu4@gmail.com; 2 Department of Electrical and Computer Engineering, College of Design and Engineering, National University of Singapore, Singapore 117576, Singapore; austin.wang@u.nus.edu (Y.A.W.); vivian.ng@nus.edu.sg (V.N.); 3 Laboratory of Intelligent Manufacturing and Micro-Nano Processing, College of Intelligent System Science and Engineering, Shenyang University, Shenyang 110044, China; yinhongyan82@163.com

**Keywords:** laser processing, micro-nano structure, functional surface, implant device, surgical instrument

## Abstract

Functional and safety requirements for medical devices are increasing with the continuous advancement of medical technology. To improve the therapeutic effect and safety of medical devices and patients, researchers are constantly exploring new materials and processes. Among them, the preparation of functional surfaces has become an important means to improve the performance of medical devices. This paper provides a comprehensive and critical review of recent advancements in laser processing technologies for the fabrication of functional surfaces in medical devices. Leveraging the unique capabilities of laser-based techniques to precisely tailor micro- and nanoscale surface structures, these methods have demonstrated remarkable potential in enhancing the therapeutic efficacy, biocompatibility, and overall safety of medical implants and surgical instruments. Such innovations are paving the way for the development of next-generation medical devices with multifunctional surface properties, meeting the increasing demands of modern clinical applications. The review focuses on the key applications, including cell function regulation, antibacterial properties, corrosion resistance, friction characteristics, and anti-adhesion properties. It also explores the considerable potential of laser processing technology, while addressing the challenges associated with multifunctional surface design and material selection. Looking ahead, the paper discusses future directions for the application of laser processing in novel materials and complex biomimetic structures.

## 1. Introduction

Precision medicine has been established as an important component of global development strategies and has received widespread and in-depth attention and recognition globally, lately, due to the increasing emphasis on people’s health and well-being [1,2]. As a key component of precision medicine, medical devices used for implantation and minimally invasive surgery (MIS) are gradually becoming mainstream due to their trauma-free, low infection risk, fast healing, and highly personalized features. Contact with tissue or organs during medical procedures is unavoidable. This can have a significant impact on patients, including biocompatibility issues with implanted devices, high-temperature coagulation with electrosurgical scalpels [3], and tissue slippage with surgical forceps [4,5,6]. As wearable devices have rapidly developed, sweaty and flexible skin can significantly reduce the device’s fixation and distort the detected signals [7,8]. Therefore, it is crucial to regulate the functional surface contact state to eliminate these adverse effects. However, the process of creating such functional surfaces is a highly challenging task of a multidisciplinary nature, involving materials, mechanical engineering, and biological sciences.

Laser processing technology is a high-precision machining method using photothermal effects. By precisely controlling the output and movement of the laser beam, it can perform various operations on various materials, such as drilling, cutting, welding, scribing, and heat treatment. The advantage of this technique is that it enables the precise construction of micro-nano structures, whether in two-dimensional or three-dimensional space. The laser-generated micro-nano structures on the surface possess the potential to alter the mechanical, chemical, tribological, and optical characteristics of the material surface [9,10,11], including but not limited to anti-icing [12], enhanced corrosion resistance [13], self-cleaning [14], enhanced Raman scattering effects [15], enhanced heat transfer efficiency [16], and reduced friction and anti-wear of mechanical components [17,18]. In the field of medical devices, lasers can realize the processing of complex and fine functional micro-nano structures, giving medical devices diversified properties. Medical implantable devices need to be biocompatible, antimicrobial, and resistant to corrosion [19]. Moreover, surgical instruments have shorter contact times with human tissues, and thus, their surface friction properties and anti-adhesion properties are more crucial. While medical materials can meet the necessary mechanical and biochemical performance requirements, their specialized surface properties often require additional treatment. Laser surface modification is an effective approach to enhance the surface characteristics of medical devices [20].

Recently, laser technology has been widely used to prepare surfaces with unique biological functions to enhance the medical performance of medical devices [21]. Table 1 shows the classification and required functions of some medical implantable devices and surgical instruments. Medical implantable devices require improved biocompatibility to promote cell adhesion, proliferation, and differentiation [22] and corrosion resistance to regulate the degradation rate [23]. They also require a certain degree of antimicrobial properties to prevent infections due to direct contact with human tissues [24]. Surgical instruments, such as surgical forceps and vascular clamps, require a higher coefficient of friction at the clamping interface to ensure stable and reliable clamping during procedures [25]. In contrast, orthopedic instruments like ball end milling cutters and drilling tools must focus on reducing wear, enhancing wear resistance, and lower machining temperatures during usage [26].

This paper aims to explore the application of laser processing technology in the field of medical devices, with a particular emphasis on the development of functional surfaces. It will focus on how laser surface modification technology affects cell function regulation, antibacterial performance, corrosion resistance, and the friction characteristics of surgical instruments, as well as aspects such as anti-adhesion. Through a comprehensive review of the current research and application status, this paper seeks to provide a clear and directional reference for the future advancement of laser surface modification technology in medical materials, thereby promoting its further development and broader application in medical devices

## 2. Potential Mechanisms of Functional Surfaces for Medical Devices

### 2.1. Fundamentals of Functional Surface Contacts

Many organisms in nature have evolved unique and delicate surface structures that perform a variety of critical contact functions. For example, the mouth edges of the pitcher plant (Nepenthes alata) and the surface of its pitcher trap exhibit super hydrophobic properties, while the toes of geckos and tree frogs have strong wet and dry adhesive capabilities, and mosquito proboscises can penetrate the skin painlessly. These surfaces can achieve special functions because they have unique material properties and micro-nanostructures that integrate complex influences of surface chemistry, fluid mechanics, and surface mechanical interactions. In-depth research into the underlying mechanisms of these functional surfaces is expected to provide innovative solutions to key challenges in the field of medical devices and drive high-performance applications.

**Figure 1:**
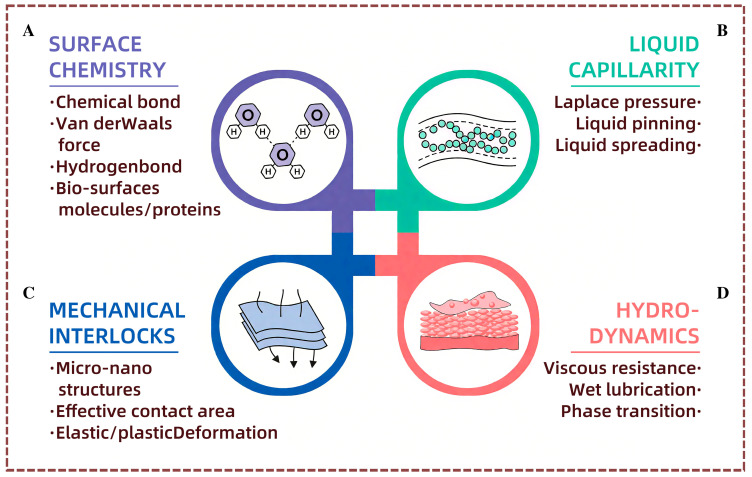
Key factors influencing interfacial contact mechanisms. (**A**) Surface Chemistry: Molecular forces affect interaction with biological surfaces. (**B**) Liquid Capillarity: Liquid behavior at interfaces influences adhesion. (**C**) Mechanical Interlocks: Micro/nano structures enhance contact and adhesion. (**D**) Hydrodynamics: Shear and pressure generate interfacial resistance.

The phenomenon of surface contact in the interactions between solid surfaces usually involves the intrinsic material properties of the solid surfaces, such as elasticity and surface chemical properties, as well as different structural forms at the micro-scale, such as columnar structures, protrusions, grooves, or random roughness. Under dry contact conditions, material properties and structures have a significant impact on adhesion performance, including adhesion force and friction. Specifically, the surface chemical characteristics have an impact on contact interactions at the molecular level through high-activity chemical groups that form strong covalent bonds or through weak hydrogen bonds or van der Waals bonds (Figure 1A). Surface structures, on the other hand, adjust adhesion performance by changing the material’s elasticity and creating or eliminating mechanical interlocks at the interface (Figure 1B). Under wet contact conditions, the liquid present at the interface becomes another key factor that determines the contact performance, with liquid properties including strong capillary action at the micro- and nanoscale, phase transitions with temperature increases, and significant fluid dynamics under shear or pressure (Figure 1C,D). These properties can regulate liquid movement or generate acting force at the interface, thereby promoting solid contact interactions and creating unique functionalities that regulate the adhesion performance. Understanding these interface contact mechanisms is of great importance for developing deep strategies for medical device design, thereby improving medical applications.

### 2.2. Anti-Adhesion and Super-Slip Properties of Natural Surfaces

**Figure 2:**
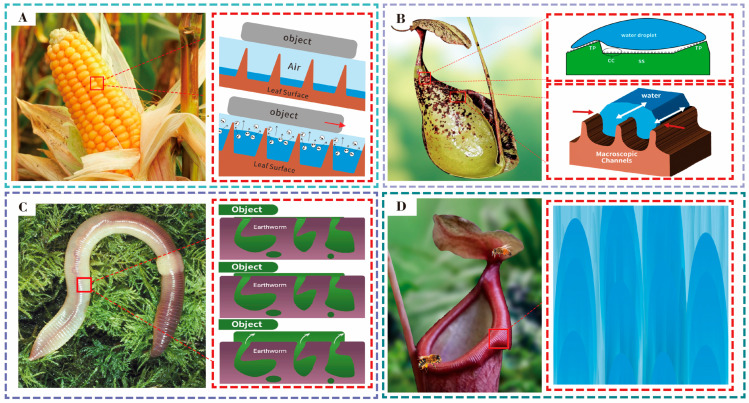
(**A**) Anti-adhesive properties of corn leaves, with longitudinal and transverse ridged structures that capture air and reduce adhesion, forming a steam cushion at high temperatures. (**B**) Slippery surface of pitcher plant traps, featuring crescent-shaped protrusions and a waxy coating that provide super hydrophobicity and lubricity. (**C**) Earthworm skin secreting viscous fluid, enabling movement through soil with long-term lubrication due to microstructural pits. (**D**) Rim surface of the pitcher plant showing rapid, directional liquid transport through microgrooves with duckbill-shaped structures, promoting super lubrication.

Certain surfaces have been found to exhibit anti-adhesive or slip properties by reducing adhesion, such as the dry contact surface of corn leaves and the moist inner wall of the pitcher plant’s trap. The surface of corn leaves is covered with longitudinal and transverse ridged structures, which help capture air between interfaces and reduce the actual contact area, thereby reducing adhesion. When the temperature rises and interface moisture evaporates, the closed grid-like ridges can generate the water vapor [27,28], further promoting contact separation and forming anti-adhesive properties at high temperatures (Figure 2A). Pitcher plants, which are carnivorous plants, have slippery inner walls of their pitcher traps that prevent the fall of insects by creating a low-adhesion surface. This surface is described as having a waxy coating and is arranged in crescent-shaped protrusions. Because of the synergistic effects of low-surface-energy materials and layering structures [29], the surface exhibits super hydrophobicity and super lubricity (Figure 2B). This unique surface structure and material are crucial for forming a non-adhesive surface under dry contacts. However, in these contact surfaces, the further reduction in adhesion is constrained by the existence of solid–solid mechanical interlocks.

Fluids with dynamic fluid properties can serve as lubricants by altering the mechanical interlocks between solids to achieve laminar fluid flow, thereby significantly reducing the adhesion strength [30]. As a result of this mechanism, the skin of earthworms secretes a layer of moist and viscous fluid to enable its movement through soil and prevent damage from solid substances (Figure 2C). In particular, due to the microstructural pits on the skin surface, this secretion can prevent the worm from being wiped off during its crawling, thus achieving a long-term lubricating effect [31]. The rim surface of the pitcher plant has evolved a functional structure that can rapidly and directionally transport nectar from the inner to the outer wall of the pitcher, to attract and capture insects into the pitcher (Figure 2D). The surface is super hydrophobic, consisting of two levels of microgrooves, with duckbill-shaped arch structures aligned at the bottom of each secondary microgroove.

These material properties can enhance the liquid expansion in one direction through a powerful wedge capillary action, while preventing the flow in the opposite direction through the pinning effect of sharp edges. This ultimately results in rapid and directional liquid transport. Through this unique function, the liquid film on the rim surface can continuously expand, keeping it in a super lubricated state.

## 3. Functional Surfaces of Medical Implants

### 3.1. Functional Surfaces for Cell Function Regulation

The surface nanostructures of implantable device materials have a significant effect on cell adhesion, proliferation, differentiation, and other behaviors, which are important influencing factors of biocompatibility. It has been found that implantable device surfaces with nanostructures are more conducive to the realization of cell function regulation than smooth surfaces [32]. Laser processing technology can rapidly prepare various micro-nano structures on the surface of implantable devices, which promotes cell adhesion, proliferation, and differentiation by changing the shape of the structures and the spacing of the arrays, to achieve the purpose of regulating cell function.

To create such micro-nano structures, laser processing may be used. Multiple studies have demonstrated the effective use of laser processing to create microgroove structures on the surface of titanium alloy materials. In the experiments, they found that the machined microgroove contact angle of the surface was reduced, resulting in an increase in the roughness value and most cell growth along the microgroove. The enhancement of hydrophilicity provided a better growth environment for cells, and the microgroove structure could significantly promote the proliferation and differentiation of mouse osteoblasts, ultimately improving their bioactivity. This discovery holds significant importance for the development of novel biomedical materials. Particularly in the field of orthopedic implants; by designing the surface microstructure, the compatibility between the material and biological tissues can be effectively enhanced, thereby improving the success rate and service life of the implants.

**Figure 3:**
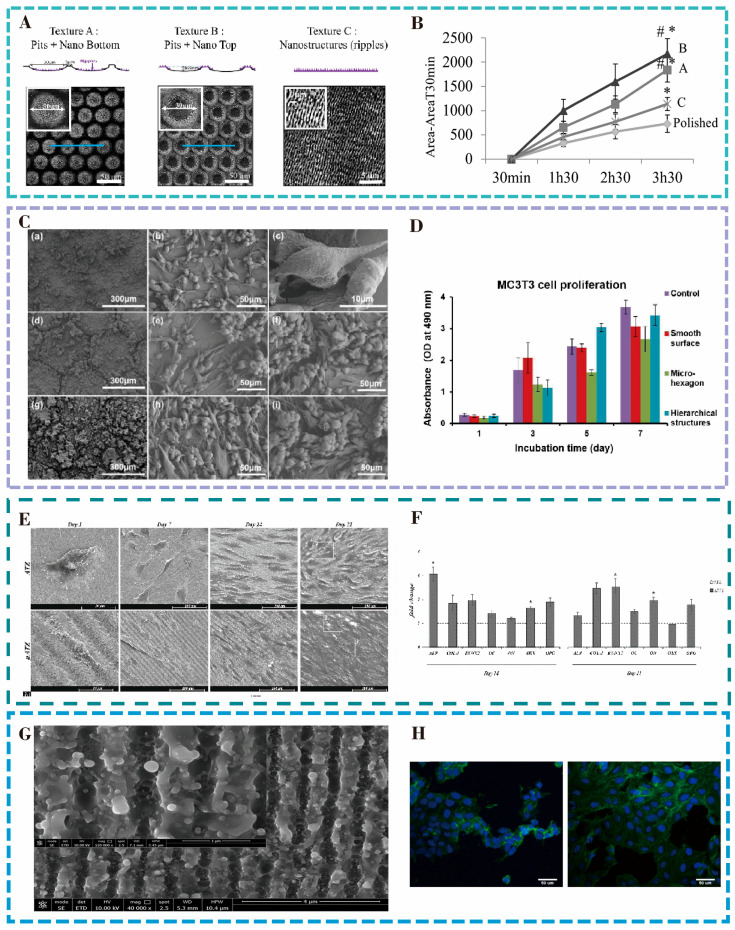
(**A**) Surface micro-nano structure of Ti6Al4V prepared by femtosecond laser. (**B**) Change in cell morphology and size over time. * *p* < 0.05 versus polished, # *p* < 0.05 versus C. Reproduced with permission of Dumas et al., 2015 [33]. (**C**) Number of MC3T3 cells on smooth, bionic hexagonal, and layered surfaces. (**a**) SEM image of MC3T3-E1 osteoblasts cultured on the smooth surface of Ti6Al4V for 7 days, High-magnification micrographs of the cells in (**b**,**c**), (**a**), (**d**) SEM image of MC3T3-E1 cells cultured on the surface of Ti6Al4V with a hexagonal array of 200 µm side length for 7 days, High-magnification maps of the interior of the hexagon and the channel area in (**e**,**f**) and (**d**), (**g**) SEM image of MC3T3-E1 cells cultured on the surface of Ti6Al4V with hierarchical structure and 200 µm hexagonal array for 7 days, High-magnification maps of the interior of the hexagon and the channel area in (**h**,**i**) (**g**). (**D**) Change in MC3T3 cell density over time. Reproduced with permission of Li et al., 2020 [34]. (**E**) Adhesion of human bone marrow mesenchymal stem cells on smooth and μATZ surfaces over time. (**F**) Expression of osteogenic transcription factors on μATZ structure. Reproduced with permission of Carvalho et al., 2018 [36]. (**G**) SEM image of HR-LIPSS on Ti6Al4V by femtosecond laser, forming ~820 nm period nano-ridges (height: 300–350 nm) with modified surface morphology and chemistry. (**H**) Fluorescence images of U-2-OS and MG63 cells on HR-LIPSS show enhanced adhesion, uniformity, and polarization versus untreated surfaces. Reproduced with permission of Iaroslav et al., 2024 [37].

Dumas et al. [33] investigated the cellular functions of mesenchymal stem cells (MSCs) on the microtextured surface of a titanium alloy using a femtosecond laser, and the shapes of the Ti6Al4V surface microstructures and their effects are shown in Figure 3A. The surface of the alloy prepared with the femtosecond laser is prepared with three kinds of surface micro-structures, respectively, including the micropits with nanoripples on the inside (Structure A), the micropits with nanoripples on the outside (Structure B), and the surface of the nanoripples (Structure C), respectively. The results of the analyzed experiments indicated that all three surface micro-nanostructures were able to significantly improve the transformation and proliferation of MSCs to osteoblasts. Under the same incubation time, the cell expansion rate of the structured surface was also much larger than that of the smooth surface, as shown in Figure 3B.

The natural organisms have developed excellent structures and morphologies, which provide a new reference and methodology for the design and development of biomimetic engineering. Li and colleagues [34] employed laser technology to fabricate a bionic hexagonal structure, akin to the paw of a tree frog, on the surface of a titanium alloy. They reduced the surface roughness of the hexagonal shape, resulting in the formation of layered micro- and nanostructures on the surface through acid etching and alkaline heat treatments. It was found that the bionic layered structure on the surface of the alloy could improve the hydrophilicity of the surface and promote the adhesion and proliferation of mouse osteoblasts, as shown in Figure 3C. When cultured for 5–7 days, the number of bionically layered cells was stable and increased significantly compared with that on the smooth surface, as shown in Figure 3D. The rough walls in the grooves between the biomimetic hexagonal structures facilitate cell adhesion and growth. Such an effect enhances the biocompatibility of titanium alloy implant devices and the integration performance of tissues.

To enhance the cell attachment, proliferation, and osteogenic differentiation of bone screws during use, Xu et al. [35] prepared three overlapping morphologies of biomimetic microstructures based on the layered structures of fish scales and shrimps on the surface of the Ti6Al4V alloy through laser processing. These nanosecond structures significantly enhance the surface roughness (Ra = 1.15 μm) and hydrophilicity (contact angle of 10°) of the material. It was found that the microtextured surface not only promotes cell adhesion and proliferative behavior on the surface but also enhances the expression of the mineralization-related genes Collagen I, ALP, and OCN. This, in turn, contributes to the formation of a good osseointegration between the material and the tissue and accelerates bone tissue healing.

Alumina-toughened zirconia ceramics have excellent biocompatibility and are often used as materials for dental and orthopedic implants. Carvalho et al. [36] used a femtosecond laser to create composite structures on alumina-toughened zirconia ceramics, featuring microgrooves with a periodicity of approximately 10 μm, overlaid with nanoripples of 300–400 nm oriented perpendicular to the grooves. Cell culture analysis indicated that human bone marrow MSCs formed a larger cell adhesion area on surfaces with micro-nano structures than on smooth surfaces, and the number of cells proliferating increased with the culture time. As illustrated in Figure 3E, the surface micro-nanostructures could regulate cell alignment and guide proliferation compared to the untreated smooth surface. The expression of osteogenic transcription factors and genes was more pronounced on surfaces with micro-nano structures (Figure 3F).

Iaroslav Gnilitskyi et al. [37] created highly regular laser-induced periodic surface structures (HR-LIPSS) on a Ti6Al4V titanium alloy through femtosecond laser technology, forming nano-ridge wave arrays with a period of approximately 820 nanometers and a height of 300–350 nanometers. The structure in Figure 3G significantly alters the surface morphology and chemical composition of the material. XPS analysis revealed that laser treatment led to a greater concentration of surface oxides, such as Al_2_O_3_, thereby enhancing the chemical stability of the surface. Cell experiments demonstrated that, compared with the untreated surface, the HR-LIPSS surface significantly enhanced the early adhesion of U-2-OS and MG63 osteoblasts, the proliferation ability within 7 days, and the production of collagen. Further observations under fluorescence microscopy revealed (Figure 3H) that the laser nanostructures promoted the uniform distribution and obvious polarization arrangement of cells. This study confirmed the effectiveness of HR-LIPSS technology in improving implant osseointegration and osteogenic induction, enhancing the biocompatibility of materials and reducing rejection reactions. Meanwhile, by investigating the effect of the picosecond laser machining of microtextures on the surface of titanium alloys on the biocompatibility of the materials, Yu [38] found that the laser-generated microgroove structures can significantly promote cell adhesion.

Laser-fabricated surface microstructures can influence cell morphology, migration, proliferation, and differentiation by regulating the surface hydrophilicity, expression of cell transcription factors, roughness, and morphological features. Surface microstructures with hydrophilicity promote the adsorption of water molecules and proteins, facilitating cell adhesion and proliferation. Moderate micron-scale roughness provides mechanical cues that enhance the expression of transcription factors and promote cell differentiation. Additionally, specific microgrooves or pits can direct the cell orientation and alter migration patterns. Using these laser-fabricated microstructure surfaces thus allows us to achieve cell function regulation.

### 3.2. Antibacterial Functional Surfaces

As most surgeries are performed in environments exposed to air and medical implantable devices acting inside the body cannot be completely isolated from bacterial contamination, there is always a risk of postoperative bacterial infection. Implanted devices themselves, surgical tools, operating rooms, and even sterilization equipment are all potential sources for bacterial contamination [39]. The incidence of postoperative infections in orthopedic implants, lately, is about 2.4%, and bacterial infections can lead to surgical failure and increased patient suffering. The surface of the implant is a preferred site for bacterial adhesion, so antimicrobial functional surfaces are particularly important for implantable devices [40,41]. It has been found that the antimicrobial properties of surfaces with micro-nanostructures are more stable and long-lasting than those of coatings, and laser surface modification has become an effective means to improve the antimicrobial properties of implantable devices and inhibit the growth of bacteria and the formation of biofilms [42].

**Figure 4:**
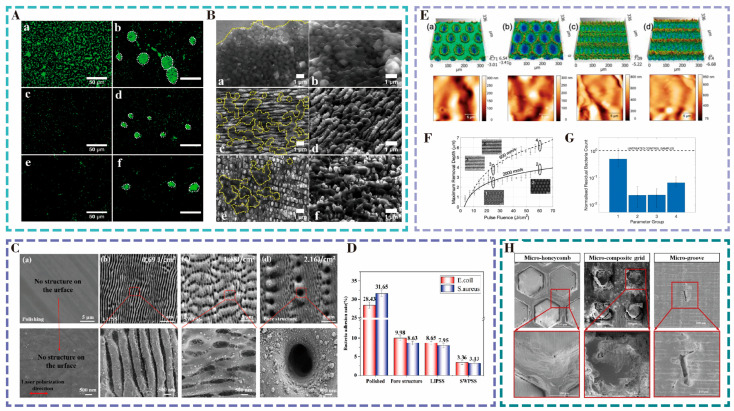
(**A**) Fluorescence images of *Staphylococcus aureus* on the structure after 48 h culture. (**a**,**b**) Polished surface, (**c**,**d**) LIPSS-structured surface , (**e**,**f**) Nanopillar-structured surface. (**B**) SEM images of *Staphylococcus aureus,* (**a**,**b**) Polished surface; (**c**,**d**) LIPSS-structured surface , (**e**,**f**) Nanopillar-structured surface. Reproduced with permission of Cunha et al., 2016 [43]. (**C**) Microstructure of zirconium-based amorphous alloy surface. (**a**) Polished surface, (**b**) LIPSS, (**c**) SWPSS, (**d**) Microholes array structure. (**D**) Bacterial adhesion rate after 24 h culture. Reproduced with permission of Huang et al., 2022 [46]. (**E**) Surface morphology of parameter groups 1–4 measured by optical profilometer (top) and scanning Hall effect microscope (bottom). (**a**) 1, (**b**) 2, (**c**) 3 and (**d**) 4 with optical profiler (top) and ShFM in central region of crater/groove (bottom). (**F**) Predicted versus experimental maximum removal depth by laser pulse energy density (solid line: 2000 mm s^−1^, dashed line: 600 mm s^−1^). (**G**) *E. coli* residue after surface treatment in different parameter groups. Reproduced with permission of Romoli et al., 2020 [47]. (**H**) SEM morphologies of 3Y-TZP materials treated with various surface textures under dry friction conditions, showing different wear characteristics and morphological changes. Reproduced with permission of Xu et al., 2022 [48].

Periodic surface nanostructures have certain advantages in the antimicrobial functionality of orthopedic titanium implants. Cunha et al. [43] used a femtosecond laser with a central wavelength of 1030 nm and a pulse duration of 500 fs generated by a Yb:KYW to induce laser-induced periodic surface structures (LIPSSs) and nano-Pillars on the surface of titanium alloy plates (TA2), with the average periods of (710 ± 60) nm and (750 ± 130) nm, respectively, and the maximum peak-to-valley distances of (250 ± 80) nm and (175 ± 40) nm, respectively. The average periods of the structures were (710 ± 60) nm and (750 ± 130) nm, and the maximum peak-to-valley distances were (250 ± 80) nm and (175 ± 40) nm, respectively. After incubating *S. aureus* on the structured surfaces for 48 h, the bacterial coverage on the LIPSS and nano-Pillar structured surfaces was much less than that on the smooth surfaces. As shown in Figure 4A,B, the size of *Staphylococcus aureus* deposits on the laser-treated surfaces was smaller under fluorescence microscopic observation. For the polished surfaces, the film was continuous and embedded bacteria were present, whereas for the laser-modified surfaces, the film was discontinuous and the sizes of the bacterial aggregates were significantly smaller.

Peter et al. [44] used the ultrashort pulsed laser system with a direct laser interference patterning technique of a 1030 nm wavelength and a pulse width of 8 ps to induce periodic micrometer structures with a period of about 850 nm and a depth of about 500 nm on a 316L stainless steel surface. The nanostructures exhibited hydrophobicity with a contact angle of (154 ± 3)°. Compared to the smooth surface, the structured surface showed a 99.8% reduction in the adhesion rate of *Escherichia coli* and a 70.6% reduction in the adhesion rate of *Staphylococcus aureus*. This is an indication that the size of the periodic micrometer structure is smaller than the size of the bacteria, which reduces the contact points between the bacteria and the surface and thus inhibits the bacterial adhesion. Meanwhile, other researchers [45] used a nanosecond Nd:YAG laser with a wavelength of 1064 nm to treat 304 stainless steel with different energy densities (5, 20, 40 J/cm^2^) to construct micro-nano structures to enhance its antibacterial properties. Among them, the hydrophobic hierarchical structure formed via 5 J/cm^2^ treatment had the best inhibitory effect on *Escherichia coli* attachment, showing significant physical antibacterial properties.

Zirconium-based amorphous alloys, a new class of biomaterials, offer superior mechanical properties compared to titanium alloys and hold significant potential for a wide range of applications. To improve the antimicrobial property of zirconium-based amorphous alloy surfaces, Huang et al. [46] performed laser path scanning on the surface of a zirconium-based amorphous alloy using a femtosecond laser with a pulse width of 300 fs and a wavelength of 1030 nm. As shown in Figure 4C, the laser induced the LIPSS structure, the super wavelength periodic surface structure (SWPSS), and the microporous structure, and then, *Escherichia coli* and *Staphylococcus aureus* were cultured on the surface of the three structures in vitro for 24 h. The results indicated that the bacteria’s adhesion rate to the three structures was significantly reduced, and the best inhibition was achieved with SWPSS (Figure 4D).

The shape of the surface structure can affect the adhesion state of bacteria, and the structure size is the key factor affecting the adhesion quantity and adhesion rate of bacteria, and when the structure size is slightly smaller than that of bacteria, the inhibition effect on bacteria is optimal.

Romoli et al. [47] utilized a nanosecond laser with a wavelength of 1064 nm and a pulse width of 104 ns to process micro-pits and micro-grooves with a depth of 3.7–6.2 μm on the surface of stainless steel 316L (Figure 4E). By conducting *E. coli* cultures, it can be observed from Figure 4F that the deeper micro-pit structure with a greater depth of micro-pits has the best antibacterial effect. In Figure 4G, it was found that the depth of the micro-nanostructure had a significant impact on the bacterial adhesion behavior, reducing the bacterial adhesion by 98%.

Xu [48] employed a picosecond laser with a power of 12 W, a wavelength of 355 nm, and a pulse frequency of 400 kHz to process the surfaces of samples for dental implants, which are susceptible to bacterial infections. The bacterial adhesion is depicted in Figure 4H. Microgroove structures with a depth of 3 μm and microhoneycomb structures with a depth of 100 μm were prepared on the surface of 3Y-TZP zirconia ceramics at scanning speeds of 1000 mm s^−1^ and 500 mm s^−1^, respectively. The cultivation of *Staphylococcus aureus* on the surface of the structures revealed that the number of colonies on the microgroove structure was higher than that on the unstructured surface, while the number of colonies on the microhoneycomb structure was lower than that on the unstructured surface, with an antimicrobial rate of about 30%. Subsequently, it was observed that the microgroove structure was hydrophilic and the microhoneycomb structure was hydrophobic. Bacteria were more likely to accumulate along the corners of the structures, and *S. aureus* was more inclined to adhere to hydrophilic surfaces, and the results indicated that hydrophobic surfaces could inhibit and reduce bacterial adhesion.

Laser-manufactured surface microtextures primarily prevent bacterial biofilms from forming on the material surface by physically blocking bacteria. Nano-pillar structures can insert or shear bacterial cell membranes, causing them to rupture and die. Periodic structures and micro-pit structures can limit bacterial aggregation by regulating the size and depth of the structures and guide bacteria to grow in a direction that leads to fracture and deformation. Many bacteria depend on aqueous environments to survive, and hydrophobic structural surfaces can reduce the time that water molecules remain on the material surface, thereby reducing the bacterial adhesion rate.

### 3.3. Corrosion-Resistant Functional Surface

When medical implantable devices are put into the body, they react to tissue fluids, such as corrosion [49]. Once corrosion occurs, dissolved metal ions and formed corrosion products can interfere with the behavior of surrounding cells, which in turn affects the microenvironment of the tissues surrounding the implant and reduces biocompatibility [50]. Corrosion behavior in the biological environment can loosen the implant, which can lead to implant failure [51]. Therefore, medical devices implanted in the human body must have good corrosion resistance in addition to high biocompatibility and antimicrobial properties if they are to meet the demands of clinical applications.

**Figure 5:**
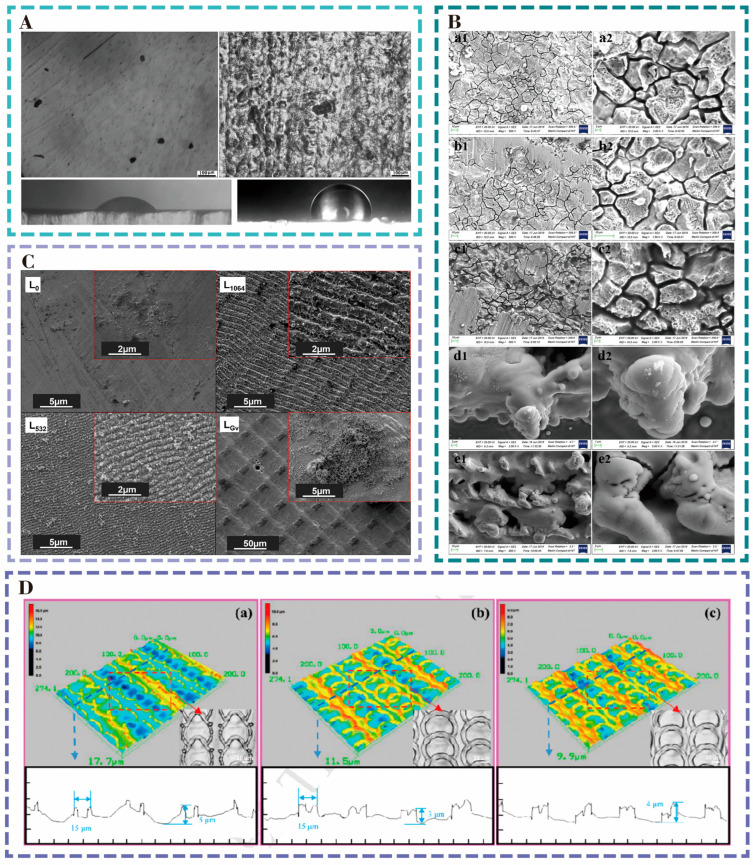
(**A**) 3D SEM images of the corroded wavy structure and laser-textured 304L stainless steel surface, showing increased hydrophobicity. Reproduced with permission of Gupta et al., 2019 [52]. (**B**) SEM images of surfaces treated at different laser energy densities with 20 mm s^−1^ scanning speed, showing texture and morphology changes with increasing energy density. (**a1**) 2.69 J/cm^2^, (**b1**) 3.96 J/cm^2^, (**c1**) 6.28 J/cm^2^, (**d1**) 8.14 J/cm^2^, (**e1**) 9.55 J/cm^2^, (**a1**–**e2**) are magnified images, respectively. Reproduced with permission of Lu et al., 2020 [53]. (**C**) SEM images after electrochemical corrosion testing for control and laser-irradiated samples, highlighting pronounced surface structures and corrosion marks. Reproduced with permission of Saqib et al., 2022 [54]. (**D**) Comparison of 3D morphology, surface roughness, and elemental analysis of laser-textured surfaces. Reproduced with permission of Xu et al., 2019 [55].

Laser processing technology as an effective means of surface modification helps to enhance the corrosion resistance properties of the surfaces of medical metal materials. Gupta et al. [52] induced a corrugated structure on the surface of stainless steel 304L using a nanosecond laser emitted by the Nd:YAG laser with a wavelength of 1064 nm, a pulse width of 100 ns, and a frequency of 2 kHz. This micro-nanostructure improved the surface roughness (Ra = 182 nm) and hydrophobicity (contact angle of 110) and was placed into a 0.5 mol L^−1^ NaCl solution for potentiodynamic polarization tests, and it was found that the surface of the corrugated structure exhibited higher pitting potentials, and the corrosion current densities were smaller by one order, as shown in Figure 5A. Meanwhile, the higher corrosion potential and lower corrosion current density indicate that the structured surfaces have higher corrosion resistance than the unstructured surfaces, and there are fewer craters formed on the structured surfaces after corrosion.

The energy density of the nanosecond laser was varied to process microcrack structures of various sizes on the surface of stainless steel 316L [53]. As shown in Figure 5B, all laser-treated surfaces showed hydrophobicity. The corrosion resistance efficiency of the surfaces was increased by more than 90% in all cases of corrosion with a 3.5% mass fraction of NaCl solution. The structure with 8.14 J cm^−2^ had the best surface corrosion resistance with a 98.61% improvement and had the highest hydrophobicity with a contact angle of 160°. To improve the corrosion resistance of vascular scaffolds, Muhammad et al. [54] laser-induced LIPSS periodic structures with a width of 220 nm and square structures with a width of 20 mm × 20 μm on the surface of stainless steel 316L (Figure 5C). Hank’s equilibrium salt solution, HBSS, corrosion results indicated that the corrosion resistance of LIPSS was nearly 50 times higher than that of the unstructured surfaces. LIPSS structures exhibit hydrophobicity and can increase the surface roughness and reduce the solid–liquid (electrode–electrolyte) contact area to decrease the corrosion rate. The LIPSS structure exhibits hydrophobicity and is able to increase the surface roughness and reduce the solid–liquid (electrode-electrolyte) contact area to reduce the corrosion rate.

Surface nanostructures can also improve the corrosion resistance of titanium alloy bone screws in service. Xu’s team [55] processed three micro-weave structures, namely the micro-bumped ring (MBR), micro-smooth ring (MSR), and micro-overlapping ring (MSSR), on the surface of Ti6Al4V using a nanosecond laser with a wavelength of 1064 nm, a pulse width of 100 ns, and a frequency of 30 kHz, as depicted in Figure 5D. Electrochemical corrosion in simulated body fluid (SBF) was performed, and it was found that the corrosion resistance of MBR, MSR, and MSSR structures was increased by 97.02%, 96.11%, and 97.97%, respectively. The results indicate that laser surface modification can significantly improve the corrosion resistance of titanium alloy surfaces. Kuczynska et al. [56] prepared two types of structures, micro-grooves and islands, on grade 2 titanium plates using a laser. Corrosion was carried out in saline at 37 °C, and it was found that both structures improved the surface corrosion resistance. The structured surfaces with greater surface roughness exhibited higher corrosion resistance, largely due to their periodic, smoother topography and the formation of a thinner, more stable passive layer. Additionally, the surface morphology played a significant role in the material’s corrosion behavior, with laser-textured surfaces exhibiting enhanced stability and resistance in corrosive environments.

Laser-prepared surface microstructures have the potential to enhance the interfacial stability between the material and the environment, thereby preventing the material surface from being corroded by chemical or electrochemical reactions. Surface microstructures can increase the actual surface area of the material surface and slow down the contact and diffusion of corrosive media with the material surface by changing the surface morphology, thus reducing the overall corrosion reaction rate.

Super-hydrophobic surfaces further inhibit the direct contact of water molecules with the metal surface, slowing electrochemical corrosion. Additionally, the laser-treated surface forms a protective oxide layer or refines the surface grains to form a remelting layer, which acts as an isolation layer and thus improves corrosion resistance.

## 4. Functional Surfaces of Surgical Instruments

The contact time of surgical instruments with human tissue during medical procedures is usually very brief, especially during surgery, where they are primarily used to perform intermittent contact operations such as grasping, cutting, and suturing. Due to the relatively short contact time, the requirements for cell adhesion and proliferation on the surface of the instruments and their resistance to corrosion are not particularly high. Nevertheless, research on their functional surfaces remains crucial. This research primarily focuses on reducing friction and wear, as well as minimizing tissue adhesion during surgical procedures. Laser surface modification technology offers a promising approach to enhancing the functional surfaces of surgical instruments. The frictional properties of surgical instruments, such as surgical knives, medical needles, and vascular clamps, can be significantly altered using laser processing, thereby not only reducing wear and extending the service life of the instruments but also improving their performance in surgery. In addition, laser surface modification can also fabricate micro-textures on the surface of minimally invasive electrosurgical devices, which can effectively reduce tissue adhesion and reduce damage to biological tissues, thereby improving the safety and success rate of surgery.

### 4.1. Functional Surfaces for Friction Regulation

Surgical instruments (surgical scalpels, puncture needles, and high-frequency electric knives, etc.) are primarily used to cut or puncture biological soft tissues, such as skin, muscle, and blood vessels, with the goal of cutting, separating, and suturing the targeted tissue area. Usually, the use of these surgical instruments does not cause adverse reactions or complications. However, in some specialized procedures, trauma caused by needle or blade friction can lead to adverse postoperative complications. For example, trauma caused by scalpel friction may trigger a cell signaling cascade response during the removal of a malignant tumor, leading to cancer cell growth or metastasis [57]. Excessive friction during puncture needle insertion can cause tissue deformation and movement, and the inhomogeneity and anisotropy of the tissue can deflect the placement of the puncture needle, leading to surgical failure [58]. During the use of vascular clips, increasing the coefficient of friction at the clamping interface helps to prevent slippage of the clip and the vessel, ensuring the safety of vascular clips. Therefore, controlling friction is of great importance in eliminating injuries that occur during the operation of surgical instruments.

**Figure 6:**
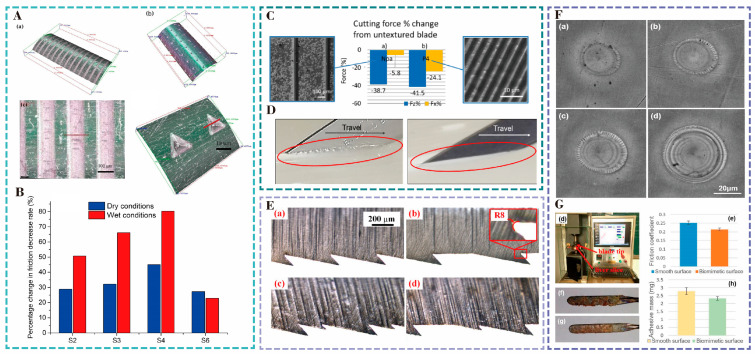
(**A**) Microscopic images of the microslot structure and cube block structure. (**a**) 3D morphology of laser-textured needle (sample S5), (**b**) Magnified view of regular dimples on S5, (**c**) Magnified view of blended dimples on S6. (**B**) Friction changes of different surface structures (S2, S3, S4, S6) in dry and wet environments. Reproduced with permission of Wang et al., 2016 [59]. (**C**) Percentage change in cutting forces (norma l force Fz and tangential force Fx) for textures generated by (**a**) DLW and (**b**) DLIP techniques compared to untreated blade. Left: DLW texture; Right: DLIP texture. (**D**) Response of polyurethane test material during cutting. Reproduced with permission of Butler-Smith et al., 2022 [62]. (**E**) Micro-serrated edge morphology of surgical blades after laser-induced periodic microstructure treatment. (**a**) type A, (**b**) type B, (**c**) type C, (**d**) type D.Reproduced with permission of Lu et al., 2020 [65]. (**F**) SEM images of surface morphology at different laser pulse energy densities, showing increasingly complex structures with higher energy densities. (**a**) 4200 J/cm², (**b**) 5000 J/cm², (**c**) 5800 J/cm², (**d**) 6600 J/cm². (**G**) Different surface properties of electrosurgical scalpels and their effects on friction coefficient and tissue adhesion. (**d**) Friction testing device for the contact between the blade and the pig liver, (**e**) Comparison of the friction coefficient between smooth and bionic surfaces (reduced by approximately 14.9%), (**f**) Image of the attached tissue after smooth blade cutting, (**g**) Images of attached tissues after bionic blade cutting, (**h**) Comparison chart of tissue quality attached to different surfaces (reduced by approximately 16.5%). Reproduced with the permission of Li et al., 2019 [64].

The laser processing of microstructures on the surface of puncture needles can effectively reduce friction. In Figure 6A, Wang [59] used a picosecond laser with a wavelength of 532 nm and a pulse of 8 ps from the Nd:YVO4 laser to prepare microgroove structures and cube-like structures with different area densities on the surface of stainless steel 304 medical puncture needles. The researchers tested the friction of different surface microstructures in wet and dry conditions and found that the friction of the puncture decreased with a decrease in the contact area. The microgroove structure with a groove width of 150 μm had the best friction-reduction effect. The friction decreased more than 80%, as shown in Figure 6B. And it was found that the microweave structure could reduce the stress concentration at the edge of the needle tip and decrease the stabbing pressure of the needle tip on the tissue [60]. For the cuboidal structure, they also found that the friction increased with the increase in the size or the number of cuboidal structures, but the depth of the structure had no significant effect on the friction. Pan [61] used a nanosecond laser to prepare four types of micro-pit structures, including circular, square, elliptical, and triangular, on the surface of 316L stainless steel. They applied them to brain electrode steel needles and performed insertion force measurement experiments on pig brains. The final results strongly suggest that the microtextured steel needles had obvious friction-reduction effects and that the elliptical micro-pit structure had the best friction-reduction performance, with an average reduction of 37.6% in friction. This effectively reduced the damage to the brain tissue.

As shown in Figure 6C, Butler used femtosecond and nanosecond lasers to machine microgroove structures on the surface of a stainless steel 316 scalpels to minimize friction during scalpel cutting [62]. They investigated the effects of the groove width and depth, groove spacing, and groove orientation on the friction properties of scalpels under dry conditions. They found that the frictional properties were significantly improved compared to the unstructured blade, with a 17.0% and 33.2% reduction in friction during cutting on the femtosecond and nanosecond laser-treated surfaces, respectively, and a smoother cut on the surface of the microgrooves, as shown in Figure 6D. Increasing the coefficient of friction at the vascular clamping interface can help prevent slippage. Nitta et al. [63] prepared microgroove and micro-pit structures on the surfaces of titanium vascular clamps using a laser and found that the microgroove structure with a groove width of 30 µm and a spacing of 40 µm had the highest coefficient of friction. The friction force was directly proportional to the actual contact area.

Shark’s skin has the function of reducing friction and antifouling, and Li et al. [64] researched shark’s skin and fabricated the bionic microtexture on the surface of a stainless steel 316L high-frequency galvanic knife using a laser. Through orthogonal tests, it was found that the bionic microtexture could reduce the effective contact area, decrease the hydrophilicity, and reduce the coefficient of friction by 21.88%. The results of the orthogonal test indicate that the microtexture plays the most important role in the friction effect. Bionic microstructures can effectively improve the surface properties of surgical instruments, as shown in Figure 6E.

Inspired by pangolin scales, their research team investigated the optimal formation parameters of laser-induced surface microstructures to prepare bionic scale microtextures on the surface of a stainless steel 316L high-frequency electrosurgical cutter, which was hydrophobic in nature. In Figure 6G, the high-frequency electric knife with bionic scales reduced the coefficient of friction by about 15% during soft tissue cutting, which effectively reduced the damage to the tissue during the procedure. Lu [65] utilized the Nd:YVO4 laser emitting a picosecond laser with a wavelength of 532 nm and a pulse width of 10 ps to ablate four serrated structures based on the edges of manzanita blades on a carbon steel scalpel blade (Figure 6E). The bionic scalpel can reduce the depth of the cut by reducing the friction between the tissue and the scalpel, thus improving efficiency and reducing tissue damage compared with the standard commercial scalpel. The magnitude of cutting force is c < a < d < b, and the friction is reduced by 19% to 44%.

The friction characteristics of medical materials and tissues can be influenced by laser-prepared surface microtextures. Appropriate surface microtextures can reduce or increase the actual contact area between the two friction surfaces, thus effectively regulating the friction characteristics. Specially shaped microtextures can reduce stress concentrations at the contact edges, decreasing the potential for tissue tearing and reducing friction. Hydrophobic surfaces can reduce friction by lowering the surface free energy and reducing the adhesion between the surface and other substances.

### 4.2. Functional Surface Against Tissue Adhesion

In clinical surgery, the electron-knife reduces pain and trauma and improves the speed of recovery after surgery [66]. The scalpel accumulates heat in a short period of time during surgery, and when the operating temperature is above 150 °C, tight tissue adhesion forms on the surface of the scalpel, and these soft tissues are difficult to remove [67], leading to such clinical adverse effects as scabs, biological contamination, and burn wounds [68]. Blood adhering to the surface of scalpel blades can cause serious adverse effects on the quality of surgery, such as delayed healing and a higher incidence of bacterial infection [69]. Therefore, anti-adhesion microsurfaces can solve the problem of blood and tissue adhesion and improve surgical safety.

**Figure 7:**
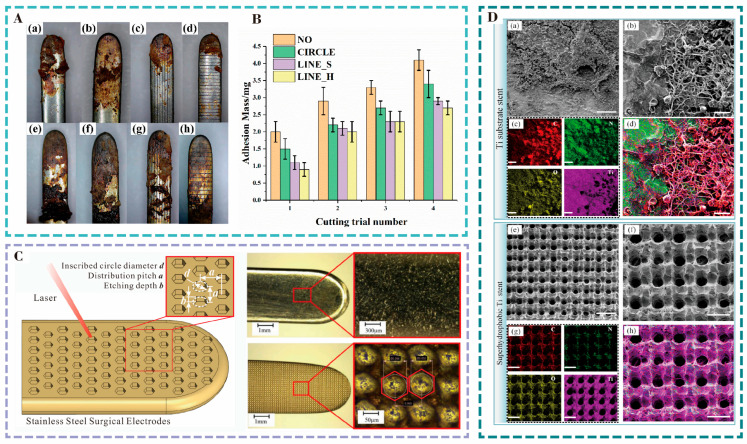
(**A**) Comparison of tissue adhesion after cutting experiments with electrodes featuring different microstructures, showing increased adhesion with repeated cuts. (**a**–**d**) are respectively the surface adhesion conditions of electrode 1 (no microstructure), electrode 2 (micro-pits), electrode 3 (longitudinal microchannels) and electrode 4 (transverse microchannels) after the first cutting, (**e**–**h**) respectively represent the surface adhesion conditions of the above four electrodes after the fourth cutting. It can be seen that the transverse microchannel structure electrodes (**d**,**h**) exhibit the optimal anti-adhesion effect. (**B**) Tissue adhesion of electrodes with different morphologies in multiple cutting experiments. Reproduced with permission of Zhou et al., 2020 [70]. (**C**) Bionic electrode design based on leafcutter ant microstructures to reduce tissue adhesion and improve surgical blade performance. Reproduced with permission of Liu et al., 2023 [73]. (**D**) Adhesion and element distribution of blood components on hydrophilic and hydrophobic surfaces, with hydrophobic surfaces significantly reducing blood adhesion and altering element distribution. (**a**) SEM image of the Ti substrate surface, (**b**) local magnified image, (**c**,**d**) element distribution map, (**e**) SEM image of the surface microstructure, presenting an ordered array, (**f**) local magnified image, (**g**,**h**) element distribution. Reproduced with permission of Zhang et al., 2021 [75].

To solve the problem of blood adhesion on the surface of the galvanic knife, Zhou et al. [70] utilized a picosecond laser with a wavelength of 532 nm and a pulse of 10 ps emitted by a Nd:YVO4 laser to process three types of surface microtextures, micro-pits, longitudinal micro-grooves, and transverse micro-grooves, with widths of 50 μm and depths of 12 μm, on the surface of the galvanic knife made of stainless steel 304 (Figure 7A). The experimental results indicated that the amount of tissue adhesion was reduced for all the microtextured electric knives. The microtexture increased the roughness of the surface of the galvanometer and decreased the actual contact area between the electrode and the tissue. Comparing the different shapes of microtextures, the transverse microgroove texture had the smallest amount of adhesion (Figure 7B), and the amount of adhesion decreased significantly with the increase in the area density of the transverse microgroove texture. Lin [71] induced nanoparticle structures with an average diameter of 100–200 nm, using a femtosecond laser, on the surface of an electrosurgical device made of stainless steel 304, which roughened the surface of the electrode and reduced the actual contact area with the tissue. It was found that the amount of tissue adhesion on the structured surface was reduced by about 70% compared to a smooth surface, thus reducing the amount of heat generated on the surface during surgery. Han [72] utilized the excellent anti-adhesion properties of corn blades at elevated temperatures to prepare a corn blade-based mesh microtexture on a stainless-steel electrode using a laser. The bionic microtexture increased the surface roughness of the electrode and reduced the contact area with the tissue. Subsequently, a TiO_2_ coating was applied to its surface using the sol-gel method, and electrocuting experiments were performed on fresh isolated animal liver tissue, and it was found that the amount of adhesion was reduced from 61 mg to 18 mg as compared with that of a normal electric knife. Based on the anti-adhesive nature of the leaf-cutting ant surface, Liu used a femtosecond laser to prepare bionic structures on a 304 stainless steel galvanic knife, as shown in Figure 7C [73]. This study found that the adhesion of the bionic structure was positively correlated with its hydrophobicity. When the structure parameters were d = 20 μm, a = 40 μm, and b = 6 μm, the bionic electrodes showed the best anti-adhesion properties, with an average reduction of about 36% in tissue adhesion compared to ordinary electrodes.

Inspired by the waterproof and self-cleaning properties of the lotus leaf surface, Li et al. [74] prepared a micro-convex circular structure on the surface of stainless steel 316L using a nanosecond laser. The surface shows strong hydrophobicity, and there is no residue on the surface after soaking in serum solution, which can effectively improve the anti-adhesion performance to serum for scalpels and vascular stents. Zhang [75] utilized a UV laser to prepare a microgrid structure on the surface of a medical pure titanium substrate, with a length of (8.8 ± 2.4) μm, a height of (22.5 ± 2.5) μm, and a slot width of (25.3 ± 0.9) μm, and chemically modified the surface of the microgrid structure to be super hydrophobic (Figure 7D). The adhesion results strongly suggested that the structured surface could effectively eliminate blood cell adhesion and inhibit thrombus formation.

Surface microtextures produced using laser technology can significantly enhance the anti-adhesion properties of medical devices. These microtextures increase the roughness of the material surface, effectively reducing the actual contact area between the tissue and the material surface. As a result, the adhesion force is greatly reduced. In addition, the hydrophobic surface formed via laser processing can reduce the contact angle with water molecules, further reducing the adhesion of tissue on the surface. This surface treatment technology not only improves the use effect of medical devices but also extends their service life, with important practical application value.

## 5. Conclusions

This review provides a comprehensive overview of the current applications and advancements of laser surface modification technology in the medical device field. We have examined the significant impact that laser processes have on regulating cellular functions, enhancing antibacterial properties, and improving the corrosion resistance of implantable devices. Additionally, we explored how laser technology can optimize the frictional characteristics and anti-adhesion properties of surgical instruments, demonstrating its potential to enhance both the performance and longevity of the instruments. Through a in-depth analysis of these studies, we summarize the parameters of different micro- and nanostructures and their key findings and draw the following conclusions:

(1) The nanostructures on the surface of the devices have a significant impact on the modulation of cellular functions, antimicrobial properties, and corrosion resistance. These effects are multifaceted and can be influenced by multiple surface properties simultaneously. With the precise design and fabrication of these micro-nano structures, it is possible to achieve coordinated modulation of these characteristics and thus optimize the performance of the devices. However, there are relatively few studies focused on designing such surfaces with composite functions, and this area remains in an exploratory phase. Although some progress has been made, we are far from achieving the level of sophistication needed to design and fabricate multifunctional surfaces. More research is needed to deepen our understanding of the relationship between micro-nano structures and device performance and to further advance the field.
(2) The field of medical devices continues to rely heavily on traditional metal materials like titanium alloys and stainless steel, which are favored for their excellent mechanical properties, corrosion resistance, and biocompatibility. However, with advancements in technology and the growing diversity of medical device needs, the exploration and application of new materials have become increasingly important. New medical device materials, such as composites, inorganic compounds, and amorphous alloys offer unique performance advantages that traditional metal materials do not have. For example, composite materials like zirconium-based amorphous alloys combine the advantages of multiple materials with good mechanical properties and corrosion resistance. Laser preparation technology also plays a key role in the functional surface treatment of these new materials, enabling the enhancement of their properties and expanding their application potential. The combination of innovative materials with laser preparation technology provides broader opportunities for the manufacture and development of medical devices and is expected to drive innovation and development in the medical device field.
(3) Bionic structures, with their unique external forms and surface morphologies, present unique properties that allow them to play a vital role in many fields. For instance, bionic structures may be applied to surfaces to provide friction-reducing benefits, minimizing friction between contact surfaces, and extend the lifespan of equipment. At the same time, the surface could also be anti-adhesive, preventing the accumulation of unwanted materials and ensuring cleanliness and efficiency in operation. Furthermore, the bionic structures can offer antimicrobial properties, which is especially significant for medical implantable devices. As these devices are in constant contact with the human body, preventing the growth of bacteria and viruses on their surfaces is critical for maintaining safety and hygiene.

As medical equipment continues to evolve towards higher performance, being minimally invasive, and personalized trends, laser processing technology, due to its non-contact nature, high precision, and good controllability, will have a broader application prospect in the field of functional surface construction. Future research should focus on the design of multi-functional integrated micro-nano structures, breaking the current limitation of “single performance regulation” and achieving the joint effect of multiple functions, such as cell regulation, antibacterial, anti-corrosion, friction reduction, and anti-adhesion on a single surface structure. At the same time, the mechanisms of the interaction between laser processing parameters (including wavelength, pulse width, scanning mode, etc.) and different materials still need to be further clarified. It is urgent to build a complete structural and functional relationship model and enhance the prediction and optimization capabilities of structural functions through simulation and big data analysis. Moreover, with the continuous emergence of new medical materials (such as amorphous alloys, composite materials, bio-ceramics, etc.), studying the structural formation laws and biological interface reactions of these materials under laser processing will become an important research direction in the future. Although biomimetic structures have many advantages, their application research in medical device surfaces is still relatively scarce. The complex morphologies of biomimetic structures on the surfaces of medical devices pose significant challenges for functional structure fabrication via laser processing. Traditional processing methods are often inadequate in achieving the required precision and complexity of such structures. Therefore, it is imperative to conduct in-depth research on laser processing technologies to enable the high-quality fabrication of biomimetic structures with intricate geometries. Biomimetic structures offer broad application prospects on medical device surfaces. To fully realize their potential and enhance the overall performance of medical devices, more systematic and in-depth studies are needed in areas such as laser fabrication methods, structural design, and functional regulation mechanisms.

## Figures and Tables

**Table 1:** Classification and required functions of some medical implants and surgical instruments.

Classification	Instruments	Application	Required Functions
Implantable Devices	Artificial Bone	Replace human bone to assist in repairing bone tissue defects	Excellent bio-compatibility; promote the growth and adhesion of bone tissue and cells
	Bone Plates + Screws	Connect and fixate, maintain the position of bones	High abrasion resistance; promote bone cell growth
	Vascular Stents	Support narrowed or occluded blood vessels, maintain patency of blood flow in the lumen	Good surface drag reduction and anti-adhesion properties, prevent restenosis
Surgical Instruments	Electrosurgical Knife	Achieve tissue separation and coagulation, serve the purpose of cutting and hemostasis	Excellent anti-adhesion properties, reduce adhesion of biological tissues due to high surface temperatures
	Surgical Forceps, Vascular Clamps	Grasp dense tissue, hold the ends of severed tissues	Provide stable grasping, strong wet friction ability to prevent slipping
	Scalpel	Used to cut skin and muscle	Low friction during cutting, reduce resistance to ensure a smooth incision
